# Analysis of Factors Related to Working Status of Dental Hygienists in Japan

**DOI:** 10.3390/ijerph18031025

**Published:** 2021-01-24

**Authors:** Hiroko Miura, Rumi Tano, Katsuo Oshima, Yoshie Usui

**Affiliations:** 1 Division of Disease Control and Epidemiology, School of Dentistry, Health Sciences University of Hokkaido, Hokkaido 061-0293, Japan; 2 Department of Health Promotion, National Institute of Public Health, Wako 351-0197, Japan; tano.r.aa@niph.go.jp; 3 Department of Dental Technology, The Nippon Dental University College at Tokyo, Tokyo 102-0071, Japan; oshima@tky.ndu.ac.jp; 4 Department of Oral Health, Faculty of Nursing and Welfare, Kyushu University of Nursing and Social Welfare, Kumamoto 865-0062, Japan; usui@kyushu-ns.ac.jp

**Keywords:** dental hygienists, employment status, early leave, oral health management, working conditions

## Abstract

The super-aged society of Japan is experiencing an increased demand for dental hygienists, of which there is currently a shortage. This study aimed to investigate the factors related to the working status of dental hygienists in Japan. We conducted a survey by mailing a questionnaire on employment to 1444 dental hygienists and obtained 537 valid responses. We conducted a bivariate analysis using either a chi-square test or *t*-test as well as a multiple logistic regression analysis to determine the factors related to working status. The overall employment rate was 68.2%, with a significant difference between age groups (*p* < 0.01). Approximately 80% of respondents considered working hours and human relations at the workplace to be important, and more than 70% of respondents considered wages as important. Finally, the following four variables were found to be significantly associated with employment status: training course attendance, a desire to work full-time, consideration of wages as important, and consideration of working hours as important. These findings suggest that it is necessary to improve working conditions and environments, including wages and working hours, as well as provide a more robust system of continued professional development for dental hygienists to increase the workforce.

## 1. Introduction

The number of teeth in the oral cavity decreases with age, and a decline in masticatory and swallowing functions, which is not seen in younger generations, is frequently observed in the elderly. In a society with a markedly aged population, it is necessary to provide new oral health services that are tailored to the oral conditions of the aged. Many studies have found that good oral health in the aged contributes to improved general health and quality of life (QOL) [1,2]. The World Health Organization (WHO) has indicated that oral health is one of the behavioral determinants of active aging [3]. Further, some epidemiological studies have reported that declining oral function may be associated with malnutrition in aged individuals [4,5]. In Japan, oral health measures for the aged play a major role in national health promotion policies, such as the establishment of goals related to the oral function of the aged in the national health plan [6]. These changes in oral health due to aging of the population have a significant impact on the work of dental professionals.

Due to the aging of the global population, Federation Dentaire Internationale (FDI) launched The Oral Health for an Ageing Population (OHAP) project in 2015 to strengthen the role of the oral health community in achieving health longevity [7]. Usui et al. conducted a survey of dental experts on areas of dental need that were expected to increase in the future in Japan. The study reported that a high percentage of respondents cited “home dentistry”, “dentistry for the aged”, “feeding and swallowing”, “regenerative dentistry”, and “preventive dentistry” as the key areas [8]. Additionally, oral care services provided regularly by dental professionals have a significant effect on reducing aspiration pneumonia [9]. The provision of oral health services is fundamental to maintaining the health of the elderly.

It has been reported that oral health care provided by dental hygienists is also effective in enhancing swallowing functions [10]. In a recent epidemiological study, the introduction of an oral function improvement program for the aged reduced the risk of frailty [11]. Thus, dental hygienists play an essential role in the implementation of oral health care services for the aged. As the number of elderly people increases, dental hygienists need to be adequately deployed in the community to ensure continued oral health care. In addition, it is necessary to supply sufficient professional education to dental hygienists concerning elderly care to provide safe oral health services to the aged with underlying diseases. In Japan, the percentage of elderly aged 65 years and above had already reached 28.4% in 2019. This is an extremely high percentage, even among developed countries [12] and, consequently, there is a great need to increase the workforce of dental hygienists.

The prevalence of dental caries in children has decreased significantly in Japan. In addition, the proportion of elderly individuals who still have their original teeth is increasing each year. According to the 2016 Survey of Dental Diseases in Japan, 51.2% of people at 80 years of age have 20 or more of their original teeth remaining. However, the prevalence of dental caries and periodontal disease among the aged remains high. Thus, there is a strong need to expand the system for providing lifelong oral health services. Furthermore, oral health services for the aged requiring long-term care are provided by the long-term care insurance system that started in 2000. When a dental hygienist under the direction of a dentist provides oral care to aged people who require nursing care, the nursing care insurance provides additional benefits. If a dentist or dental hygienist provides technical advice and guidance on oral care to nursing staff working in a residential facility, they can also receive additional payment under the nursing care insurance system.

As of 2018, the total number of employed dental hygienists in Japan was 132,635, of which only 73 were men. In Japan, the Dental Hygienist Act granted dental hygienist licenses to mainly women until 2013. As a result, 99.95% of employed dental hygienists were women. There is a significant shortage of dental hygienists who can provide adequate dental health services to all generations [13]. Thus, there is an urgent need to expand the workforce of dental health hygienists. However, the ratio of employed dental hygienists to the total number of registered dental hygienists was merely 46.0% in 2018 [14]. To address the shortage of dental hygienists in Japan, it is important to establish work policies that allow registered dental hygienists to continue working. However, few studies have explored the employment status of dental hygienists in Japan.

In addition to basic conditions of employment, such as wages and working hours, related factors such as marriage and childbirth may affect employment since most dental hygienists in Japan are women. Furthermore, acquiring adequate professional education for rendering dental hygiene services is another factor affecting employment status. Therefore, the purpose of this study was to investigate factors related to the working status of dental hygienists in Japan by comparing the multiple factors between employed and unemployed dental hygienists.

## 2. Subjects and Methods

### 2.1. Study Setting

This study was a self-administered questionnaire survey for members of three alumni associations of dental hygiene training schools; it was not a national survey. Since all members of the alumni association were women, no gender analysis was conducted in this study. The distinction between unemployed and employed was based on whether the respondents were engaged in dental hygiene work at the time they answered the questionnaire. Those who were engaged in some occupation but not engaged in dental hygiene work were classified as not employed.

### 2.2. Selection of Subjects and Research Design

The study included three alumni associations representing dental hygienist training schools in three different regions (Kanto, Kyushu, and Hokkaido) of Japan. All three alumni associations have been in existence for more than 40 years. We conducted a survey using a self-administered questionnaire to individuals aged 20–59 between August 2018 and December 2019. Since only 4.5% of employed dental hygienists in Japan are over 60 years old, the target age range for analysis was defined. The questionnaires were sent to 1444 alumni members by mail, and the secretariat of each alumni association executed the stratified random sampling method. The survey organizer received stickers printed with the sampling results, which were used to send the questionnaire to the alumni members. The respondents completed the questionnaires anonymously.

### 2.3. Survey Items

Based on the national survey items concerning employment [15], we examined the following: the subjects’ basic characteristics, employment status, working condition preferences, and participation in professional skill development training programs within the past year. We obtained basic demographic information such as the respondents’ age, marital status, and number of children. The respondents were asked about their current and desired employment status (full-time or part-time). The respondents were also asked to indicate whether they assign priority to each of the following five items using a binominal scale: wages, working hours, working location, job description, and human relations at the workplace.

### 2.4. Analysis Methods

After examining the descriptive statistics of each variable, we explored the associations between employment status and each variable by conducting a bivariate analysis using either a chi-square test or unpaired *t*-test. Next, we conducted a multiple logistic regression analysis (likelihood ratio, forward–backward stepwise selection method) to include the independent variables that showed a significant association to employment status in the bivariate analysis. We obtained the odds ratios, which were adjusted for the inter-relationship between the independent variables. We also included the alumni association category as an independent variable in the analysis since we incorporated responses from three different alumni associations in this study. Finally, we identified the factors that were closely related to the employment of dental hygienists in Japan after a multiple logistic regression analysis. The significance level was set at *p* = 0.05. IBM SPSS Statistics for Windows Version 26.0 (IBM Corp., Armonk, NY, USA) was used for statistical processing.

### 2.5. Ethical Approval

This study was conducted as per the guidelines of the Declaration of Helsinki. The analytical data did not contain any information by which the participants could be identified. The ethics review committee at the Nippon Dental University College at Tokyo approved the study (Approval No.: Tokyo Tanrin-218).

## 3. Results

### 3.1. Respondents’ Demographic Information

**Figure 1:**
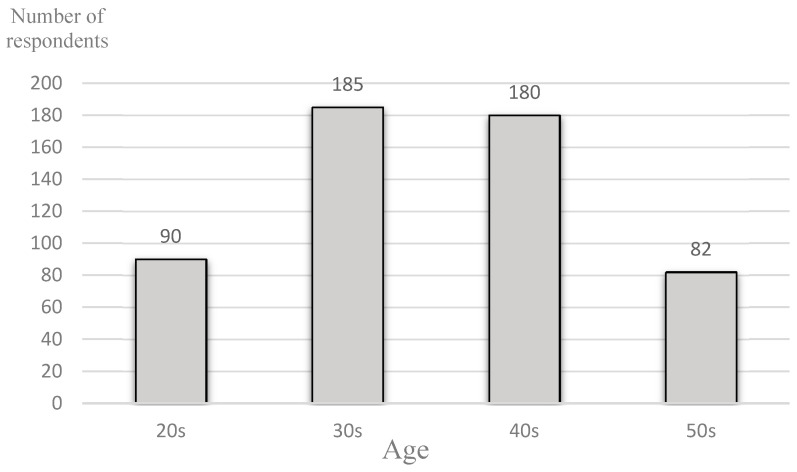
Age distribution of subjects (*N* = 537).

Of the 549 questionnaires collected, 537 valid responses were received (response rate: 37.2%). The age distribution of the 537 dental hygienists is shown in Figure 1. Approximately 66% of the respondents were in the 30- to 40-year-old age group. The mean age of the sample was 39.1 years (standard deviation: 8.9).

Table 1 provides the demographic information of the respondents related to age distribution. The employment rate was 85.6% for those in their 20s but decreased significantly to 67.6% for those in their 30s. The employment rate remained almost unchanged for those in their 40s but decreased over time to 50.6% for those in their 50s. The rate of subjects seeking full-time work followed a similar trend to the employment rate. Moreover, nearly 70% of respondents in their 30s were married.

Similarly, 65.8% of respondents in their 30s reported having children. Of the respondents in their 20s, 60% had participated in a training program, however, this rate decreased as the respondent age increased. Overall, 66.3% of respondents were married, 60.5% had children, 49.5% wanted to work full-time, and 41.5% had participated in a training program within the past year.

### 3.2. Analysis of the Priority of Working

Table 2 shows results related to variables that were prioritized by the subjects. Specifically, it provides information on the factors that the respondents selected as being important for their continued employment. More than 80% of the respondents listed “working hours” and “human relations at the workplace” to be crucial, and approximately 73% prioritized “wages”. However, 60.89% and 52.33% of the respondents prioritized working location and job description, respectively. These rates were relatively low compared to the results for other variables.

### 3.3. Analysis of Factors Related to Employment Status (1): Bivariate Analysis

We conducted a chi-square test to analyze the current employment status of dental hygienists (Table 3). The results indicated that there were significant differences between the employed and unemployed groups for the following variables: marital status, having/not having children, desire to be employed, participation in skill development training programs, and age (*p* < 0.01). The results also indicated significantly higher values in the employed group concerning the rate of seeking full-time work and participation in training programs. However, the unemployed group showed significantly higher values for marital status, having children, and age.

We also used a chi-square test to analyze the relationship between employment status and employment priorities (Table 4). The results indicate that there were significant differences between the employed and unemployed groups with regard to wages (*p* < 0.01) and working hours (*p* < 0.05). The employed group had significantly higher values for the variables concerning essential labor elements such as working hours and wages. However, there were no significant differences between the two groups in terms of working location, job description, or human relations at the workplace.

### 3.4. Analysis of Factors Related to Employment Status (2): Multivariate Analysis

Given that the bivariate analysis indicated that age might be a potential confounding factor, we conducted multiple logistic regression analysis to determine which factors affect employment status after adjusting for confounding factors (Table 5). The dependent variable was “employment status as a dental hygienist (employed/unemployed)”. Eight independent variables were included in the analysis: age, marital status, having children, participation in skill development training, the priority assigned to wages, and priority assigned to working hours. The bivariate analysis indicated that these eight variables were significantly related to employment status. We determined that the following four items were significantly associated with employment status using the multiple logistic regression analysis: “participation in skill development training programs”, “desired employment status”, “priority assigned to wages”, and “priority assigned to working hours”. However, “age”, “marital status”, and “the presence of children” were not extracted from the multiple logistic models.

## 4. Discussion

### 4.1. Trends in Employment Rate and Other Factors by Age Group

The results indicated that the employment rate sharply declined from the 20s age group to the 30s age group. Subsequently, the employment rate gradually decreased with age. The Japan Dental Hygienists Association reported a similar trend in 2017 [14]. According to a nationwide survey on overall women’s employment status in Japan [16], the employment rate declined when women were in their 30s and then increased after they reach their 40s when they no longer required childcare. However, the results of the present study indicated that the employment status of dental hygienists differs significantly from this trend. Our findings suggest that the re-employment of dental hygienists does not progress as rapidly as it does in the overall population. One explanation for this may be that dental hygienists find it challenging to maintain their dental professional skills after leaving their job. While there have been three training centers established in Japan as part of a project funded by the Ministry of Health, Labour, and Welfare to support the re-employment of dental hygienists [17], the number of training centers falls significantly short of the demand for re-employment.

### 4.2. Analysis of Factors Related to Employment

#### 4.2.1. Bivariate Analysis

The bivariate analysis revealed significant differences in demographics, such as age, marital status, having children, and desire to work full-time between employed and unemployed dental hygienists. Several previous studies have noted a similar trend [13,18]. Approximately 90% of dental hygienists in Japan worked in private dental clinics in 2018; however, most of these clinics do not have on-site childcare facilities. Thus, the social infrastructure needed to support the work–life balance of dental hygienists with children is still lacking.

In this study, the top three most prioritized factors concerning employment were the following: “working hours”, “human relations at the workplace”, and “wages”. However, there was no significant difference in the “human relations at the workplace” variable between the employed and unemployed dental hygienists. This result suggests that “human relations at the workplace” does not determine employment. In general, job description is a significant factor in choosing an employer; however, there was no significant difference between the two groups in terms of working location or job description in this study. Although some new tasks, such as oral health care for older adults, have been developed, there are few differences in the tasks that are regularly performed at common dental clinics. This is a possible explanation as to why the number of dental hygienists who prioritized the importance of job description was relatively small.

Participation in skills training programs was also related to employment status. Training institutes in Japan and Korea previously tended to emphasize dental assistance rather than dental health guidance [19]. Japan’s educational system for dental hygienists has increased the length of the required educational period since 2010 [20]. However, dental hygienists in their 30s and older have received prior education that emphasized dental assistance. In the future, job description would be of increased importance for continuous employment, depending on the increase in dental hygienists trained in the new curriculum. It is necessary to develop realistic educational programs based on dental hygienists’ actual work situations following graduation [21].

#### 4.2.2. Multivariate Analysis

The multiple logistic regression analysis set “employment status” as the dependent variable, adjusted for confounding factors such as age, and identified the following four variables as relevant factors: “participation in skill development training”, “desire for full-time work”, “priority assigned to working location”, and “priority assigned to working hours”. In a previous study [12], life events such as marriage and the delivery of a child, which have been frequently cited as factors related to employment among dental hygienists, were not extracted after adjusting for confounding factors using multiple logistic regression analysis. Our findings will be useful for increasing the number of working dental hygienists. It is also noteworthy that “participation in skill development training” and “priority assigned to wages” were extracted as variables related to employment status. It has been important for dental hygienists to have their personal, family, and public needs simultaneously met to maintain continuous employment as a dental hygienist. Participation in training programs that aim to improve professional skills should be a fundamental condition for maintaining their professional careers.

Furthermore, wages, desired employment status, and working hours are common motivational factors for all workers. Dental hygienists’ professionalism is based on intrapersonal, interpersonal, and public professionalism [22]. The variables extracted in this study consisted of three domains of professionalism. Furthermore, a path analysis of work ethic factors reported that the intrapersonal factor was closely related to work ethic among employed dental hygienists [23]. There is an urgent need to foster these three professionalism factors in dental hygiene education.

The findings obtained in the present study suggest that it is necessary to provide opportunities for self-improvement besides wages, working hours, and employment status. It is also necessary to frequently update knowledge and specific skills that were not included in traditional education curricula to provide adequate oral health care for older adults.

It is also highly likely that the working environment surrounding female dentists is similar to that of dental hygienists. A recent study reported that the burden of childcare for female dentists in Japan is so heavy that 57.1% of female dentists in Japan would like to change their working style [24]. A similar analysis of employment-related factors among female dentists in Japan should be conducted in the future.

### 4.3. Professional Education for Dental Hygienists

A noteworthy aspect of this study is that professional education played a significant role in the employment of dental hygienists. Professional education has had a great impact on enhancing the identity of dental hygienists. A study on the professional role of dental hygienists in Finland reported that there was a lack of alignment between the traditional role of the dental hygienist and the evolving scope of dental practice [25]. Even though the systems for providing oral health and dental health care services are vastly different in Japan and Finland, both countries have common educational challenges. Reassessment involving the stakeholders and improvement of current training programs would be necessary in Japan as well. In addition, a competency analysis of dental hygiene work would greatly enhance the work capacity of dental hygienists and thus ensure a larger workforce [26]. Unfortunately, there is not enough competency analysis on dental hygiene work in Japan; there is an immediate need to promote such research.

### 4.4. Provision of Oral Health Services in Japan

In Japan, the disease structure of dental diseases is changing drastically due to the rapid decline in the prevalence of childhood caries as well as the increase in the number of aged people who still have most of their original teeth. However, sufficient oral health services are not yet available for all aged people, owing to the shortage of dental hygienists. The number of dental hygienists employed in long-term care facilities accounted for only 0.3% of the total number of dental hygienists in 2018. The number of employed dental hygienists is vastly insufficient for the increasing rate of elderly people requiring long-term care.

In providing oral care services to elderly people who require nursing care, medical knowledge of the physical functions of the elderly and a welfare mindset are also necessary. For dental hygienists to continue to work, there is a strong need to expand professional education programs that foster appropriate oral care skills.

### 4.5. Limitation of This Study

As this study focused on a local population, it is difficult to analyze the national situation based on the findings from this study alone. Additionally, another limitation of this study is the relatively low response rate. Despite the limitations, this study’s multiple logistic regression analysis clarified the factors related to the employment status of dental hygienists in a wide range of age groups. We elucidated the differences in each independent variable between employed and unemployed dental hygienists. In addition, many of the items used in the survey were taken from the Labor Force Survey conducted by the Japanese government and, therefore, might have been conceptualized insufficiently.

## 5. Conclusions

The findings obtained in this study suggest that to increase the dental workforce in Japan, it is necessary to enhance working conditions and environments such as wages and working hours, as well as to provide a more robust educational system to strengthen the professional development of dental hygienists.

## Figures and Tables

**Table 1:** Demographic results by age.

Variable	Age				χ^2^-Value	*p*-Value
	20s	30s	40s	50s		
Employment rate (%)	85.55	67.56	68.33	50.62	20.93	<0.01
Marriage rate (%)	23.33	70.81	76.67	81.48	38.56	<0.01
Subjects with children (%)	14.77	65.76	71.51	76.83	36.30	<0.01
Training participation rate (%)	60.00	43.78	34.63	31.71	16.82	<0.01
Subjects seeking full-time work (%)	80.00	44.32	47.22	32.93	21.57	<0.01

**Table 2:** Prioritized variables of the subjects according to the survey results.

Variable	%
Working hours	80.82
Human relations at the workplace	80.07
Wages	73.37
Working location	60.89
Job description	52.33

**Table 3:** Difference between unemployed and employed respondents along with the five indicators.

(A) Chi-square Test				
Variable	Employed (*N* = 366)	Unemployed (*N* = 171)	χ^2^-Value	*p*-Value
Marriage rate (%)	58.47	82.46	32.27	<0.001
Subjects with children (%)	54.10	73.68	18.32	<0.001
Training participation rate (%)	54.92	12.87	83.5	<0.001
Subjects seeking full-time work (%)	60.38	12.87	102.44	<0.001
**(B) Unpaired *t*-test**				
**Variable**	**Employed (*N* = 366)**	**Unemployed (*N* = 171)**	***t*** **-Value**	***p*** **-Value**
Age (years)	37.99 ± 8.95	41.31 ± 8.37	4.07	<0.01

**Table 4:** Relationship between employment status and employment priorities.

Variable	Employed (%)	Unemployed (%)	χ^2^-Value	*p*-Value
Working hours	83.61	74.85	5.21	<0.001
Human relations at workplace	80.33	79.53	0.01	NS
Wages	80.05	59.06	25.40	<0.001
Working location	62.57	57.31	1.18	NS
Job description	54.10	48.54	1.30	NS

**Table 5:** Relevant factors concerning employment status using multiple regression analysis.

Independent Variable	*β*	SE	Wald	*p*-Value	OR	95% CI
Participation in training programs	1.87	0.27	47.62	<0.001	6.50	3.82–11.07
Desired for full-time work	1.20	0.19	40.34	<0.001	0.30	0.21–0.44
Priority assigned to wages	0.75	0.24	9.55	<0.01	2.12	1.32–3.42
Priority assigned to working hours	0.65	0.29	5.11	<0.05	1.91	1.09–3.34
Constant	1.14	0.43	6.97	<0.01	3.13	

*β* = beta coefficient, SE = standard error, Wald = Wald χ2 statistics, OR = odds ratio, CI = confidence interval.
